# Profuse Bleeding From a Pyogenic Granuloma As the First Clue to von Willebrand Disease in a Pediatric Patient

**DOI:** 10.7759/cureus.89677

**Published:** 2025-08-09

**Authors:** Brian A Moreno, Ziad Khatib, Ana Duarte

**Affiliations:** 1 Department of Dermatology, Lake Erie College of Osteopathic Medicine, Bradenton, USA; 2 Division of Neuro-Oncology, Nicklaus Children’s Hospital, Miami, USA; 3 Division of Dermatology, Children's Skin Center, Nicklaus Children's Hospital, Miami, USA

**Keywords:** academic dermatology, clinical dermatology, complex dermatology, dermatology care, dermatology consult, dermatology screening, general dermatology, inclusive dermatology training, pediatric dermatology, pediatric dermatology emergency

## Abstract

A 12-month-old female presented with a friable, hemorrhagic papule on the right lateral inferior eyelid, clinically consistent with a pyogenic granuloma. Although pyogenic granulomas are known to bleed, the extent of hemorrhage in this case was unusual and occurred after minor trauma. The patient’s father, an emergency medicine resident, injected lidocaine with epinephrine and applied pressure at home to control the bleeding. Notably, the patient's mother has a history of von Willebrand disease, prompting concern for an underlying inherited bleeding disorder. The severity of bleeding led to referral to pediatric hematology for further evaluation, including hemoglobin and hematocrit testing. This case highlights an uncommon presentation of a common lesion and emphasizes the importance of recognizing disproportionate bleeding from a common skin lesion as a potential early sign of an undiagnosed coagulopathy in pediatric patients.

## Introduction

Pyogenic granulomas (PGs) are common benign vascular proliferations that typically develop in response to cutaneous trauma, irritation, or hormonal changes. Despite their name, they are neither pyogenic nor true granulomas; histologically, they represent lobular capillary hemangiomas with a predilection for rapid growth and frequent bleeding due to their fragile vasculature [1]. PGs are particularly prevalent in children and young adults, often presenting on the face, trunk, or extremities, and though generally self-limited, they can provoke concern due to their friability and tendency to bleed [2].

While minor bleeding is a well-documented characteristic of PGs, cases involving excessive or recurrent hemorrhage should prompt further evaluation for an underlying hemostatic disorder. In this case, the patient’s maternal history of von Willebrand disease (VWD) heightened clinical suspicion following an exaggerated bleeding event. VWD is the most common inherited bleeding disorder, with an estimated prevalence of up to 1% in the general population. It results from a quantitative or qualitative defect in von Willebrand factor (VWF), a glycoprotein essential for platelet adhesion and stabilization of factor VIII [3]. Type 1 VWD, the most frequent subtype, is characterized by partial quantitative deficiency of VWF and often presents with mucocutaneous bleeding, easy bruising, and prolonged bleeding following minor trauma or procedures [4].

Pediatric patients with subtle or isolated symptoms, such as unusual bleeding from a common lesion, may go undiagnosed without a high index of suspicion. Laboratory workup typically includes assessment of VWF antigen, VWF ristocetin cofactor activity, and factor VIII levels. Interpretation requires consideration of age, stress, blood type, and recent bleeding episodes, all of which can influence circulating VWF levels [5,6]. A thorough personal and family history is also critical, as mild VWD can present with non-specific signs and may only be unmasked during a hemostatic challenge [7]. In some cases, cutaneous findings, such as excessive bleeding from a benign lesion, may be the first clinical clue to an inherited bleeding disorder.

This report highlights an unusual clinical presentation in which an exaggerated bleeding response from a PG in a 12-month-old child led to the identification of laboratory findings consistent with type 1 VWD. It underscores the importance of recognizing disproportionate bleeding in pediatric dermatologic lesions as a potential clue to an underlying systemic bleeding disorder.

## Case presentation

**Figure 1:**
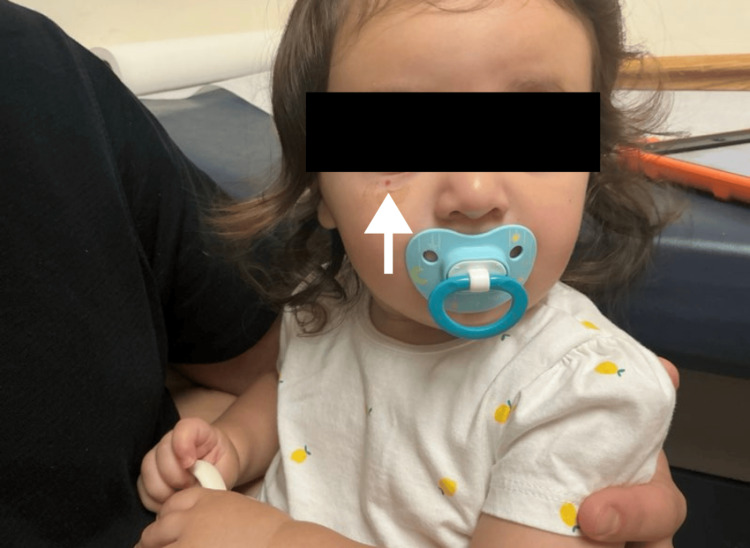
Bleeding skin lesion located on the right lateral inferior eyelid

**Figure 2:**
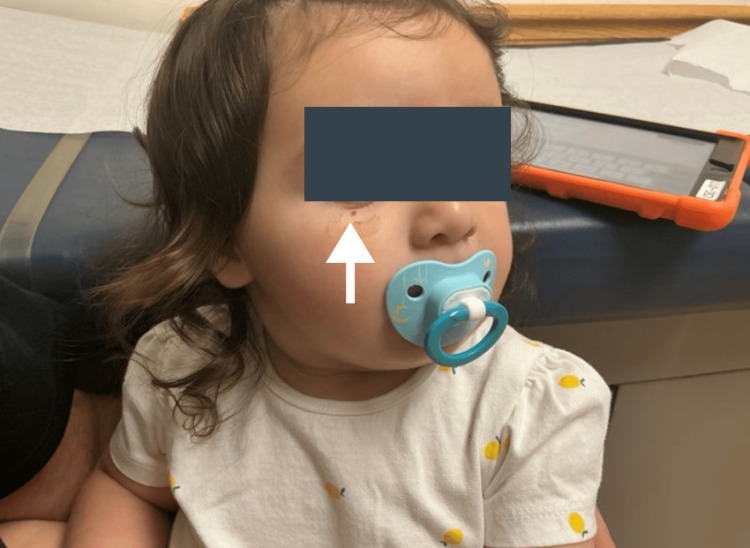
Bleeding skin lesion located on the right lateral inferior eyelid

**Figure 3:**
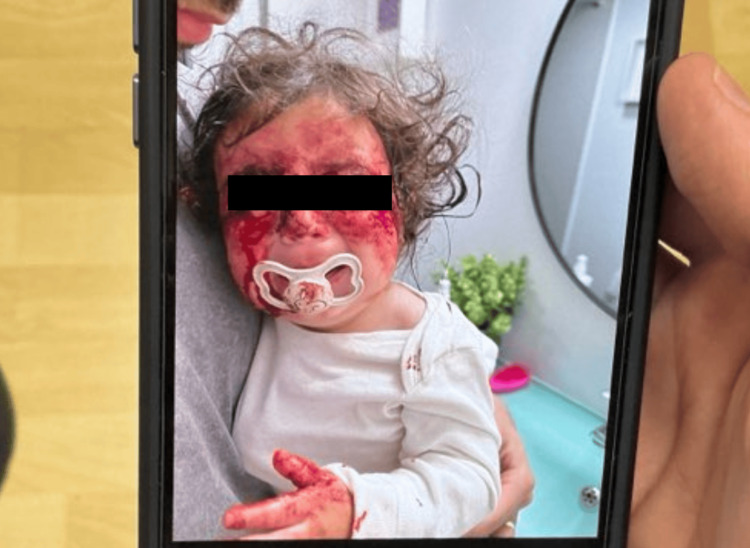
Active bleeding from PG on the right lateral eyelid following minor trauma PG: Pyogenic granulomas

**Figure 4:**
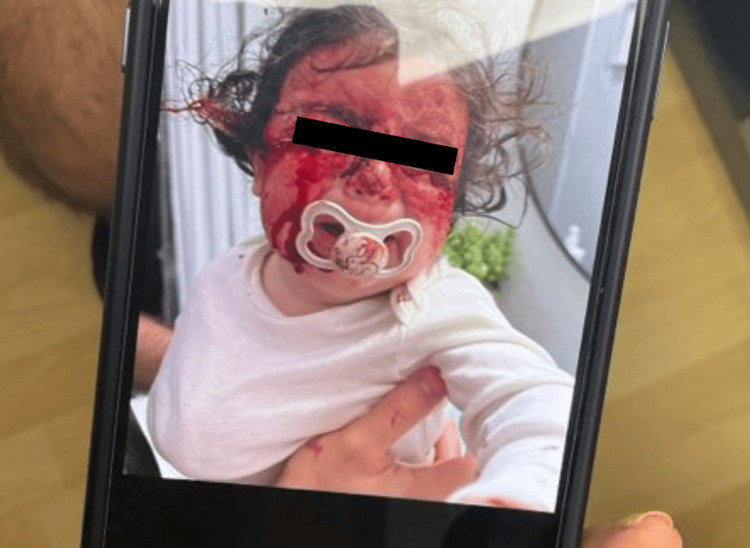
Active bleeding from PG on the right lateral eyelid following minor trauma PG: Pyogenic granulomas

A 12-month-old female presented for evaluation of a bleeding skin lesion located on the right lateral inferior eyelid (Figures 1, 2). The lesion had been present for several months, with recent worsening. Ten days prior to presentation, the patient scratched the lesion, resulting in significant bleeding that required intervention at home by her father, an emergency medicine resident, who injected lidocaine with epinephrine and applied pressure to control the hemorrhage (Figures 3, 4). The patient’s mother has a known history of von Willebrand disease, prompting concern for an inherited bleeding disorder. No prior episodes of mucosal bleeding, excessive bruising, or abnormal bleeding after minor trauma were reported by the parents. On examination, the child appeared well-developed and well-nourished, with a normal mood and affect. Skin examination revealed a friable, hemorrhagic papule consistent with a pyogenic granuloma.

Given the profuse bleeding and family history, the decision was made to pursue hematologic evaluation to rule out an inherited bleeding disorder. While the lesion itself was consistent with a pyogenic granuloma, the magnitude of bleeding was not typical for such a lesion. Initial labs revealed a normal INR of 1.0, a prothrombin time of 11.1 seconds, and an aPTT of 32 seconds. However, fibrinogen activity was decreased at 143 mg/dL (reference: 180-383 mg/dL). This sample was collected after the bleeding episode, which may influence acute-phase reactants such as fibrinogen. A von Willebrand reflexive panel showed a VWF activity of 42%, VWF antigen of 60%, and factor VIII activity of 35% (Table 1). The VWF activity-to-antigen ratio was normal at 0.7, and multimer testing was not indicated. Pathologist interpretation supported a possible diagnosis of type 1 VWD, given the clinical context and laboratory findings.

**Table 1:** von Willebrand reflexive panel results and fibrinogen activity with pediatric reference ranges (collected post-bleed episode) VWF: von Willebrand Reflexive

Test	Result	Reference Range
VWF activity	42%	55–200%
VWF antigen	60%	55–200%
Factor VIII activity	35%	50–150%

Based on these findings, the patient was referred to both hematology and plastic surgery. Recommendations included close follow-up and possible repeat testing of VWF levels, as acute-phase reactants and recent bleeding can transiently lower results. The family was advised to maintain protective covering of the lesion and to use Gelfoam sponges in the event of recurrent bleeding. Anticipatory guidance addressed the benign nature of PGs and the possibility of surgical excision, pending hematology clearance. At the time of manuscript submission, the patient had not yet returned for follow-up testing or surgical intervention.

## Discussion

Pyogenic granulomas are typically benign, self-limiting lesions that pose minimal clinical concern aside from their tendency to bleed with minor trauma. In most pediatric cases, they are managed with simple interventions such as shave excision, electrocautery, or pulsed dye laser therapy, and bleeding is usually controlled with local measures [1]. However, when bleeding is profuse, prolonged, or recurrent, especially in the absence of ongoing trauma, it is critical to consider the possibility of an underlying coagulopathy. This case illustrates how a routine dermatologic presentation can serve as an early signal of a systemic disorder, such as von Willebrand disease (VWD). Although bleeding is common with PGs, profuse or prolonged hemorrhage is unusual. Published series quantifying bleeding severity in PGs with underlying coagulopathies are limited, and more systematic data would be helpful.

VWD is the most common inherited bleeding disorder, affecting up to 1% of the general population, though many individuals remain undiagnosed due to its variable clinical expression [3]. The diagnosis is particularly challenging in infants and young children, where overt bleeding episodes may not yet have occurred. In this patient, the history of excessive hemorrhage from a relatively minor lesion, combined with a maternal history of VWD, appropriately raised clinical suspicion. Although von Willebrand disease is inherited in an autosomal dominant manner, this was the child’s first significant bleeding episode, which prompted initial testing. Laboratory results revealed reduced von Willebrand factor activity (42%), antigen (60%), and factor VIII activity (35%), which are findings consistent with type 1 VWD in the setting of clinical bleeding [3,4].

The diagnostic process for VWD requires careful interpretation. Levels of von Willebrand factor can fluctuate based on stress, illness, or recent bleeding, and are known to be lower in individuals with blood group O [5]. In this case, the recommendation for follow-up testing reflects best practice per current guidelines, which emphasize the need for repeat evaluation once acute bleeding has resolved to confirm a stable diagnosis [3,6]. Fibrinogen was also mildly decreased (143 mg/dL), which may have contributed to the bleeding tendency. This value may reflect an acute-phase response or transient consumption; however, repeat testing would help distinguish a transient effect from mild hypofibrinogenemia. Although this isolated fibrinogen reduction is nonspecific, it reinforces the importance of a comprehensive coagulation workup in pediatric patients with unexplained bleeding. While platelet count and morphology were not assessed in this case, these would also be important in evaluating superficial bleeding, especially when platelet function disorders are a consideration.

Importantly, the identification of suspected type 1 VWD in this case prompted not only patient-specific intervention but also initiated a broader family discussion regarding screening and recognition of bleeding symptoms. The early diagnosis carries meaningful implications for future procedures and trauma management. This case has since influenced internal practice guidelines in our dermatology clinic by prompting the inclusion of family bleeding history in the assessment of patients presenting with vascular lesions and atypical hemorrhage. 

Given the autosomal dominant nature of VWD, genetic counseling may be beneficial for affected families. While the patient had a known family history of VWD, this case is notable for being the first clinical manifestation in the child. The excessive bleeding from the pyogenic granuloma served as the initial diagnostic clue, influencing both patient management and internal clinic screening practices.

This case underscores the need for heightened clinical awareness of inherited bleeding disorders in pediatric dermatology. While the findings from a single case cannot be generalized, it demonstrates the diagnostic value of listening closely to clinical history and considering systemic causes of localized symptoms. Seemingly benign skin lesions may offer the first visible clue to a deeper hematologic diagnosis [2,7].

## Conclusions

This case highlights the importance of considering an underlying bleeding disorder when faced with atypical or profuse bleeding from a commonly encountered skin lesion, such as a pyogenic granuloma. In this 12-month-old patient, significant hemorrhage following minor trauma, combined with a positive family history, led to the identification of laboratory findings consistent with type 1 von Willebrand disease. Early recognition allowed for timely hematologic referral and appropriate care coordination. Clinicians should maintain a high index of suspicion for inherited coagulopathies in pediatric patients with disproportionate bleeding, as dermatologic presentations may serve as the first clinical indicator of systemic disease. This case reinforces the value of considering systemic diagnoses even in common dermatologic presentations and demonstrates how early recognition of atypical bleeding patterns can uncover undiagnosed coagulopathies.
